# Experimental Investigation on the Bending Performance of Steel–Concrete Composite Beams After Creep

**DOI:** 10.3390/ma18235332

**Published:** 2025-11-26

**Authors:** Faxing Ding, Yang Dai, Xiaolei He, Fei Lyu, Linli Duan

**Affiliations:** 1 School of Civil Engineering, Central South University, Changsha 410075, China; dinfaxin@csu.edu.cn (F.D.); daiyang0909@163.com (Y.D.); lyufei@csu.edu.cn (F.L.); linliduan@csu.edu.cn (L.D.); 2 CHN Energy Baoshen Railway Group, Baotou 014010, China

**Keywords:** steel-concrete composite beam, creep, shear connection degree, flexural bearing, internal force redistribution, concrete strength degradation, cross-sectional shape

## Abstract

The long-term flexural performance of steel-concrete composite beams after creep is influenced by multiple factors such as the degree of shear connection, cross-sectional form, and boundary conditions. The engineering community has an ambiguous understanding of the coupling effects of these factors. To address this issue, this paper conducts systematic experimental research: six simply supported beams (three box-shaped, three I-shaped) and four continuous beams (two box-shaped, two I-shaped) were designed with three degrees of shear connection (0.57, 1.08, 1.53). These beams first underwent simulated creep tests (24 °C, 80% relative humidity, 10 kN load, 180 days), followed by monotonic bending tests. The results indicate: (1) A high degree of shear connection (1.53) reduces creep deflection by 15–20% compared to partial connection (0.57) and delays the initiation of interface slip to 30% of the ultimate load; (2) Box sections exhibit 10–15% lower creep deflection than I-sections, though both experience 40–60% stiffness reduction after creep; (3) Continuous beams show a 25% improvement in crack resistance in the negative moment region and a 50% increase in flexural capacity at mid-span compared to simply supported beams; (4) After creep, the elastic modulus of concrete decreases by 40–60% (inversely related to the degree of shear connection), with fully connected specimens retaining 55–61% of their strength, while partially connected specimens retain only 43–49%. This study quantifies the degradation patterns of concrete performance, clarifies the influence mechanisms of key structural factors, and provides theoretical and experimental support for the long-term performance design of composite beams. Shear connection design is crucial for mitigating creep effects.

## 1. Introduction

Composite structures have emerged as a critical form in modern engineering construction, and are widely used in many cutting-edge fields such as aerospace [1,2], civil engineering [3,4,5], marine engineering [6,7], and transportation infrastructure [8,9] owing to their advantages of high strength, fire resistance, excellent ductility, strong energy dissipation capacity, and construction convenience. These structures typically take the form of components such as slabs, beams, and columns. Composite structural members effectively combine steel and concrete, which are two materials with complementary advantages [10,11,12,13]. This combination significantly mitigates the inherent limitations of each material used alone and achieves a synergistic enhancement effect [14,15], consequently attracting extensive research attention [16,17,18,19,20,21]. Particularly in building and bridge engineering, reliable shear connectors enable synergistic interaction between steel and concrete, allowing full utilization of the high tensile strength of steel and the high compressive strength of concrete [22,23,24,25,26], thereby markedly improving overall structural performance and economic efficiency.

Research on composite beams has a long history, dating back to the early 20th century. In 1922, Mackay H.M. [27] from the University of Toronto conducted the first test on steel-concrete composite beams, which took the form of steel beams encased in concrete. Around the same time, the National Physical Laboratory in the UK carried out similar investigations. Early studies primarily focused on fire protection and did not incorporate shear connectors. Subsequent research demonstrated that interface slip could lead to a significant reduction in load-carrying capacity. During the 1950s, Viest [28] and Thürlimann [29] conducted systematic research on stud shear connectors. They highlighted the considerable contribution of the tensile yield strength of studs and the elastoplastic deformation of concrete to the effective load-bearing capacity, and were the first to propose an empirical formula for the critical strength of shear connectors. Furthermore, in 1954, Viest established a calculation formula for the bearing capacity of studs and their critical slip through push-out tests [30]. Subsequently, research gradually expanded to include continuous beams, partial shear connection, and construction stages, among other areas. Johnson et al. [31] proposed a computational model for studs based on systematic experimental studies. Research on composite structures in China also began in the 1950s. The Design Code for Steel and Timber Structures of Highway Bridges and Culverts, issued in 1974 [32] and its revised edition in 1986 [33] clearly specified the structural detailing and calculation methods for composite beams.

During the 1970s to 1980s, research on composite structures continued to deepen, and international design standards such as Eurocode 4 [34] fully incorporated relevant design methods. After 1978, a research team led by Professor Nie Jianguo at Tsinghua University achieved groundbreaking results in the mechanical behavior, stiffness calculation, and prestressing applications of composite beams [35,36,37,38,39], advancing both theoretical understanding and practical implementation. During the same period, the emergence of new structural forms such as corrugated steel webs [40], concrete-filled steel tubular composite beams, and trough-shaped composite beams significantly enhanced the lightweight potential, construction efficiency, and mechanical performance of composite beams.

Since the 1990s, research has further expanded to the innovation and performance analysis of shear connectors. In addition to traditional studs, perfobond rib shear connectors have received significant attention due to their superior fatigue resistance [41]. The performance degradation of stud connectors under corrosion and cyclic loading has become a key research focus [42]. In terms of bearing capacity and stiffness calculations, researchers have found that the traditional transformed section method underestimates actual deformations. Consequently, various improved stiffness reduction methods and finite element models that consider shear slip effects have been proposed to more accurately reflect the actual structural behavior [43]. Dynamic studies have revealed that the natural vibration frequency of composite beams decreases due to interface slip, exhibiting a significant stiffness reduction effect. Furthermore, the dynamic response demonstrates a non-monotonic relationship with static stiffness [44,45,46,47].

Since the beginning of the 21st century, research on composite beams has continued to expand into multiple areas, encompassing the influence of concrete creep on stress redistribution [48], the torsional behavior of curved composite beams under seismic action [49], the effects of environmental temperature and humidity on creep [50], as well as the calculation of prestress loss and fatigue performance in externally prestressed composite beams [51], among others. These studies have significantly enriched the design theoretical framework of composite beams and have been widely applied in numerous bridge and building structures, such as the Wuhan Yangtze River Bridge [52], the Taiyuan First Thermal Power Plant [53], the Shenzhen China Resources Center [54], and the Beijing LG Tower [55]. The technology of achieving synergistic interaction between steel and concrete through shear connectors has been fully validated in modern engineering structures due to its excellent mechanical performance.

**Figure 1:**
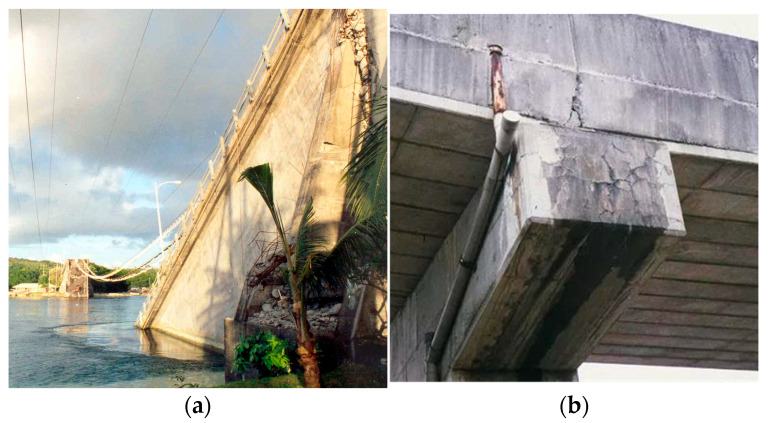
Case Study of Bridge Deflection Due to Creep Effects: (**a**) The Koror-Babeldaob Friendship Bridge. Source: https://chinesedrywall.wje.com/projects/detail/koror-babeldaob-bridge (accessed on 10 May 2025; (**b**) The Huangshi Yangtze River Bridge. Source: https://www.ztcjjt.com/a/xwzx/hyzx/20240615/7505.html (accessed on 10 May 2025).

However, despite the increasing application of composite beams in modern structures and the abundance of research investigating their short-term mechanical behavior-covering parameters such as cross-sectional dimensions and geometry, concrete type and strength, and loading conditions-studies on their long-term performance, particularly behavioral changes induced by concrete creep effects, remain relatively limited. This research gap partly stems from the long-standing focus of both academia and engineering practice on composite columns, especially their critical role in high-rise buildings and seismic engineering. Nevertheless, the creep effect of concrete under sustained loading leads to significant internal force redistribution, adversely affecting the mechanical performance and service life of composite beams. As illustrated in Figure 1, two typical failure cases exemplify this phenomenon: the Koror-Babeldaob Bridge in Palau [56,57,58], which developed a mid-span deflection of 1.61 m after 18 years of operation and collapsed two years later; and the Huangshi Yangtze River Bridge in China [59,60], with a main span of 245 m, completed in 1995, which exhibited a deflection exceeding 300 mm after only seven years of service. These examples demonstrate that neglecting creep effects may obscure potential risks during long-term service.

Indeed, significant progress has been made in research on concrete creep. Gilbert R.I. and Tarantino A.M. [61,62] established a theoretical framework for time-dependent creep analysis of composite structures, and Bradford M.A. [63] revealed the fundamental performance characteristics of continuous composite beams through long-term loading tests. However, the mechanisms of material property evolution under extreme environmental conditions remain to be further investigated. Moreover, existing methods still have limitations in accounting for material nonlinearity and interfacial slip behavior. Domestic scholars have also conducted systematic research: Fan Lichu [64] proposed the initial strain method and energy principles to analyze internal force redistribution caused by creep; Fan Jiansheng [65,66] improved the accuracy of the equivalent cross-section method through long-term experiments; Li Faxiong [67] developed a shrinkage and creep analysis module to enhance computational efficiency; and Zhao Guanyun [68] optimized reliability assessments via stochastic factor analysis. Although these achievements are notable, the applicability and accuracy of these methods in continuous composite beam systems still require further validation. The simplified time-dependent analysis method proposed by Zdeněk P. [69] is computationally efficient but does not fully consider material nonlinearity and interfacial slip, limiting its engineering applicability under complex loading conditions.

Currently, with the continuous advancement of major infrastructure projects worldwide, the long-term performance of composite structures has become increasingly critical. For example, the Humen Bridge exhibited fatigue cracks in the steel box girder welds after 27 years of operation, and the specialized maintenance project implemented following the vortex-induced vibration incident in 2020 [70,71] cost 280 million yuan, including the use of carbon fiber composites to repair damaged areas, installation of tuned mass dampers, and upgrades to the main cable dehumidification system. The under-construction Ya’an-Nyingchi section of the Sichuan-Tibet Railway [72,73,74] (2021–2030) faces even more severe environmental challenges: an average altitude of 3500 m and an annual extreme temperature difference of 60 °C. Consequently, the project employs technical measures such as nano-modified concrete [75,76], lead-core isolation bearings [77,78], and a comprehensive monitoring system along the entire line, with a total investment of 850 million yuan. These engineering practices not only demonstrate the excellent performance of composite structures [79,80,81,82,83,84,85] but also reflect the challenges they face in long-term service under complex environmental conditions [86,87].

Based on the aforementioned research background and engineering needs, this paper conducts a systematic experimental investigation. However, despite the urgency of addressing related engineering problems, significant research gaps remain in the current academic understanding of the long-term performance of steel–concrete composite beams. Existing studies have predominantly focused on short-term mechanical behavior or the influence of single parameters, lacking a comprehensive analysis of the flexural performance of composite beams after creep, particularly regarding the coupled effects of factors such as cross-sectional shape and boundary conditions. Furthermore, the quantitative relationship between the degradation of concrete material properties (such as elastic modulus and compressive strength) induced by creep and the overall structural response remains unclear. There is also insufficient experimental evidence concerning the internal force redistribution in continuous composite beams under creep effects and the cracking behavior in the negative moment regions. Current design methods do not adequately account for the long-term influence of creep on structural stiffness and bearing capacity, which may lead to deviations in performance evaluation in practical engineering applications.

To address these research gaps, this study sets the following objectives: (1) to investigate the influence of the degree of shear connection, cross-sectional shape (box versus I-section), and boundary conditions (simply supported versus continuous) on the flexural performance of composite beams after creep; (2) to quantify the degradation laws of the elastic modulus and compressive strength of concrete after creep and establish a correlation model with the degree of shear connection; (3) to clarify the characteristics of internal force redistribution and the evolution mechanism of crack resistance in the negative moment regions of continuous composite beams under creep effects; and (4) to propose calculation methods for the stiffness and bearing capacity of composite beams that consider creep effects, thereby providing theoretical and experimental support for engineering design.

To achieve these objectives, a series of tests was carried out. First, monotonic loading tests were conducted on three simply supported steel–concrete composite box beams and three I-beams after long-term performance testing, to analyze the effects of creep on their bearing capacity, stiffness, and degree of shear connection, as well as to examine the influence of cross-sectional shape. Subsequently, based on the stiffness reduction method and back-analysis of experimental data, the degradation laws of the elastic modulus and compressive strength of concrete after creep were revealed. Finally, static loading tests after creep were performed on two unequal-span continuous composite box beams and two I-beams to study the evolution mechanisms of key parameters such as stiffness and bearing capacity. This research aims to provide systematic experimental evidence and theoretical support for the design and safety assessment of the long-term performance of steel–concrete composite beams.

## 2. Materials and Methods

### 2.1. Explanation of Key Symbols

Table 1 lists the core symbols involved in the design, testing, and analysis of steel-concrete composite beam specimens in this study, including stiffness parameters, material property parameters, and shear connection characteristic parameters. These symbols will be frequently used in subsequent sections (such as specimen parameter design, creep test procedures, and result analysis), and their specific definitions and application scenarios will be further elaborated in conjunction with the context to ensure clarity in the description of test methods and results.

### 2.2. Specimen Design

The design of composite beam specimens in this study was centered on the Code for Design of Steel Structures (GB 50017-2017) [88], focusing on two key factors. All parameters were comprehensively determined based on engineering practice scenarios, experimental research objectives, and relevant specification requirements to ensure the rationality of specimen structure and the reliability of test results, laying a foundation for subsequent creep and static loading tests.

#### 2.2.1. Degree of Shear Connection (*η*)

As a core indicator reflecting the collaborative working ability between steel and concrete, the degree of shear connection is defined by Equation (1):

(1) $$\eta =\frac{\eta_{r}}{\eta_{f}},$$

where $\eta_{r}$ denotes the actual number of shear connectors installed, and $\eta_{f}$ denotes the number of shear connectors required for full shear connection design. Composite beams can be classified into two categories—full shear connection and partial shear connection—based on the number of studs.

To cover the common range of shear connection degrees in engineering and systematically explore its mitigation mechanism on structural performance degradation caused by creep, three typical levels of shear connection degree were selected in this study: 0.57 (partial connection), 1.08 (full connection), and 1.53 (η≥1.0 over-connection). This classification strictly adheres to the provisions of the Code for Design of Steel Structures (GB 50017-2017), where full shear connection is defined as and partial shear connection as η<1.0. Precise control of different shear connection degrees was achieved by adjusting the spacing of studs.

#### 2.2.2. Cross-Sectional Form of Steel Beams

The steel beams of composite beam specimens adopted two typical cross-sectional forms: box-section and I-section. Both are the most widely used cross-sectional types for composite beams in bridge and construction engineering, with strong engineering representativeness, enabling effective reflection of the mechanical characteristics of different cross-sectional forms in practical engineering.

To eliminate the interference of cross-sectional stiffness differences on test results and ensure the comparability of mechanical properties between the two cross-sections, their dimensions were designed following the principle of equivalent cross-sectional moment of inertia: the box-section steel beam was determined to have a height of 80 mm and a width of 260 mm, while the I-section steel beam had a height of 160 mm and a flange width of 100 mm. This design ensures that the basic stiffness of the two cross-sections remains comparable, thereby focusing on the influence law of the cross-sectional form itself on the bending performance of composite beams after creep.

**Figure 2:**
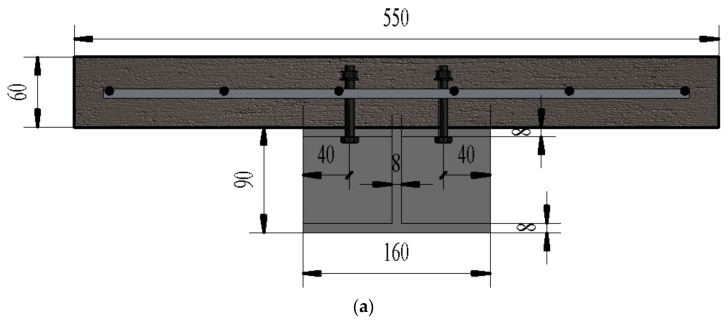

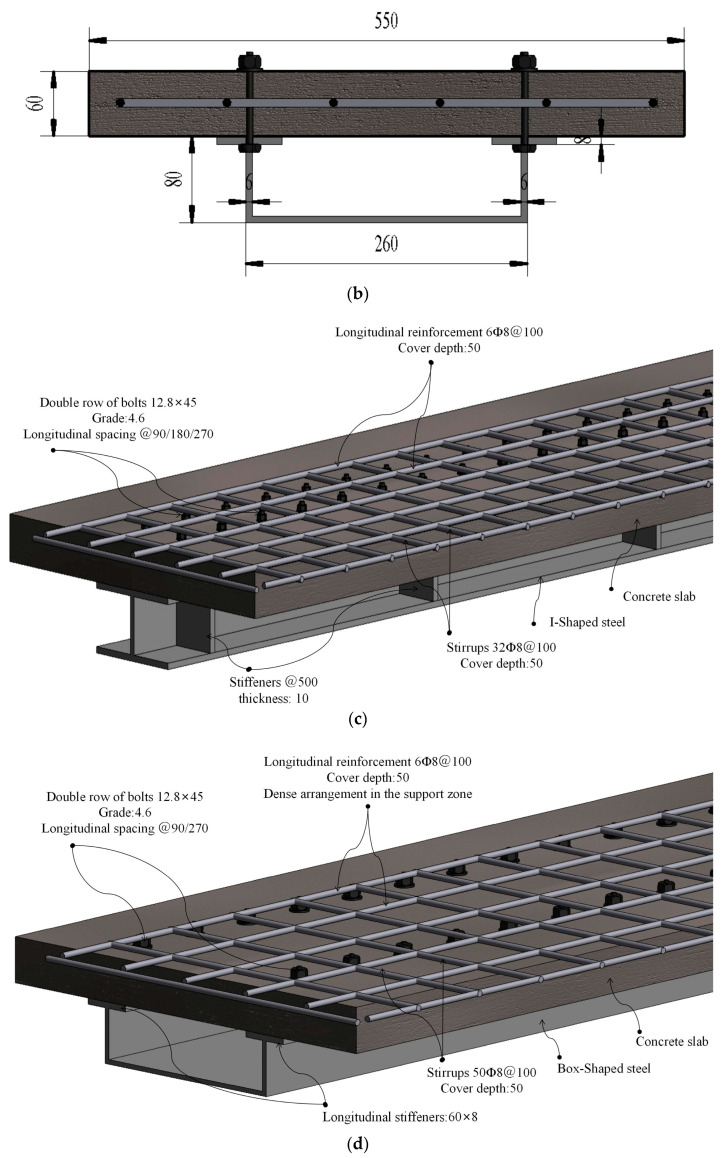
Specimen design drawing (mm): (**a**) I-shape cross-section; (**b**) Box-shape cross-section; (**c**) I-shape cross-section 3D diagram; (**d**) Box-shape cross-section 3D diagram.

The simply supported steel-concrete composite beams and continuous steel-concrete composite beams in the test maintained consistent basic structures, with differences only in net span length and number of specimens: the simply supported beams had a net span of 3000 mm, and the continuous beams had a total span of 5000 mm (adopting an unequal span of 3 m + 2 m). The core dimensions of the steel beams and concrete slabs were completely uniform for both types of beams, with specific design details as follows:

1. Span Design: Span parameters were determined with reference to engineering examples of medium- and small-span composite beams (e.g., urban footbridges, floor beams in industrial plants). The design of a 3000 mm net span for simply supported beams and a 5000 mm total span (3 m + 2 m unequal span) for continuous beams not only adapts to the range limitations of the test loading equipment to ensure stable and controllable loading processes but also realistically simulates the actual stress state of composite beams under common spans in engineering, enhancing the engineering reference value of test results.
2. Steel Beam Structure: The box-section steel beam had dimensions of 80 mm (height) × 260 mm (width), and the I-section steel beam had dimensions of 160 mm (height) × 100 mm (flange width). To improve the local stability of the steel beams and avoid premature local buckling of the steel beams during the test (which would affect result accuracy), 10 mm-thick diaphragms were installed every 500 mm along the length of the steel beams. Meanwhile, longitudinal stiffeners were added to the bottom of the box-section steel beams to further enhance their anti-buckling capacity.
3. Concrete Slab and Reinforcement: The concrete slab was designed with a thickness of 60 mm and a width of 550 mm, with HRB335 grade Φ8@100 steel mesh embedded inside. This configuration conforms to the conventional engineering practice for reinforcing the tension zone of flexural members in composite beams-the steel mesh can effectively bear the tensile force in the tension zone of the concrete slab, preventing a sudden drop in bearing capacity after concrete cracking, while strengthening the collaborative working effect between the concrete slab and the steel beam to ensure they participate in force bearing together.
4. Shear Connectors: Grade 4.6 studs with a diameter of 12.8 mm and a length of 45 mm were selected as shear connectors. In simply supported beams, three stud spacings (90 mm, 180 mm, and 270 mm) were set to simulate shear connection degrees of 0.57, 1.08, and 1.53, respectively; in continuous beams, two stud spacings (90 mm and 270 mm) were used to represent full shear connection and partial shear connection, respectively. Grade 4.6 studs are the mainstream type of shear connectors in engineering, and their diameter and length were determined based on the shear force transfer requirements of composite beams; the stud spacing was calculated according to the designed shear connection degree and verified against the requirements for construction accuracy and spacing deviation in the Code for Manufacture of Railway Steel Bridges (TB 10212-2009) [89] to ensure the fabrication quality of specimens meets engineering standards.
5. The cross-sectional forms and reinforcement details of the beams are shown in Figure 2, and the specific parameters of all specimens are listed in Table 2, which can provide complete basic data support for subsequent creep tests, static loading tests, and result analysis. In addition, strict control was imposed on material quality and construction accuracy during specimen fabrication: all specimens were made of Grade II reinforcing steel and Q235 grade steel beams; commercial C40 concrete was used, with a measured compressive strength of 34.5 MPa; the entire fabrication process complied with the requirements of the Code for Manufacture of Railway Steel Bridges (TB 10212-2009) [89] to ensure all specimens meet quality standards and have good performance consistency, avoiding the impact of individual specimen differences on test result accuracy.

### 2.3. Experimental Procedures

**Figure 3:**
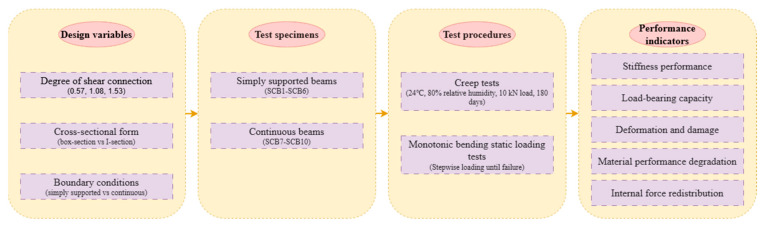
Research methodological framework for composite beam creep and flexural tests.

To clarify the logical chain of the experimental methodology, Figure 3 presents the research framework, spanning from design variables to performance evaluation.

This framework systematically links design variables (shear connection degree, cross-sectional form, boundary conditions), test specimens (simply supported and continuous beams), test procedures (creep test and static loading test), and performance indicators (stiffness, load-bearing capacity, etc.), providing a clear roadmap for the experimental process.

#### 2.3.1. Creep Test of Composite Beam Specimens

Creep tests on all specimens were conducted in a fully enclosed environment, equipped with precision hygrometers and thermometers to record daily changes. The test environment was maintained at approximately 24 °C with a relative humidity of about 80% to ensure stability. A symmetrical load of 10 kN was applied to the distribution beam, and the experiment was performed in the Creep Laboratory of the School of Civil Engineering at Central South University.

**Figure 4:**
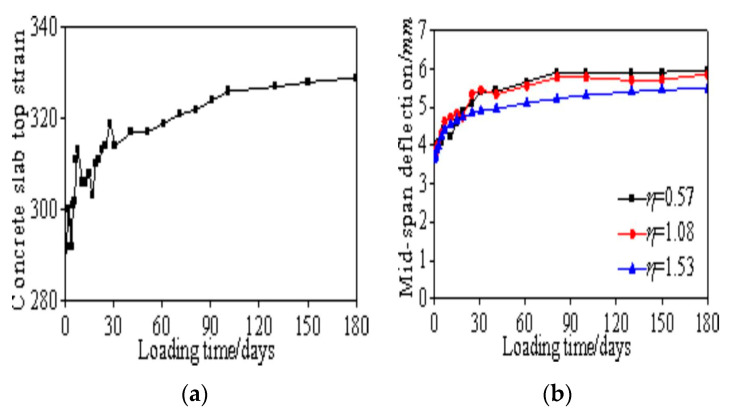
Creep behavior curves of composite beam specimens: (**a**) Midspan deflection curves of box-shaped composite beams; (**b**) Strain curves of the concrete top slab for specimen SCB1.

The test began with a concrete age of 28 days and consisted of four stages: preloading, load adjustment, formal loading, and unloading. Measurements were taken of the midspan deflection, steel beam strain, and concrete slab strain. Due to the low applied load, the compressive stress in the concrete slab was minimal. The midspan deflection curves for box-shaped composite beams with different shear connection degrees over time are shown in Figure 4a, indicating rapid initial deflection changes that stabilized after 150 days. Higher shear connection degrees resulted in smaller creep-induced deflections. The stress–strain curve for the concrete slab of specimen SCB1 is shown in Figure 4b, with a maximum compressive strain of 290. Calculations yield $f_{c}=24.89\;\mathrm{Mpa}$, $E_{c}=30,926\;\mathrm{Mpa}$, $\sigma =\varepsilon \times E_{c}=8.97\;\mathrm{Mpa}$, and $\sigma /f_{c}=0.36$, confirming that the stress level remains within the elastic range.

#### 2.3.2. Static Test Loading Scheme

**Figure 5:**
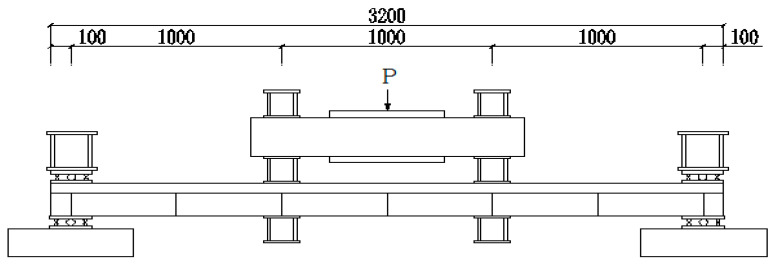
Schematic Diagram of Monotonic Loading Plan for Composite Beams.

All simply supported and continuous composite beam specimens underwent forward monotonic static loading tests at the National Engineering Laboratory for High-Speed Railway Construction Technology. Precise load control was achieved using hydraulic jacks equipped with force sensors, with specific configuration details illustrated in Figure 5. Rigid pads measuring 1000 mm×1000 mm×1000 mm (Figure 4) were installed at support and loading points to effectively prevent concrete crushing and steel beam buckling. Supports were leveled with cement mortar and fixed to reaction beams anchored to the ground, completely restraining lateral displacement. Throughout testing, load and displacement data were continuously recorded in real-time to provide foundational data for generating load-deflection curves.

**Figure 6:**
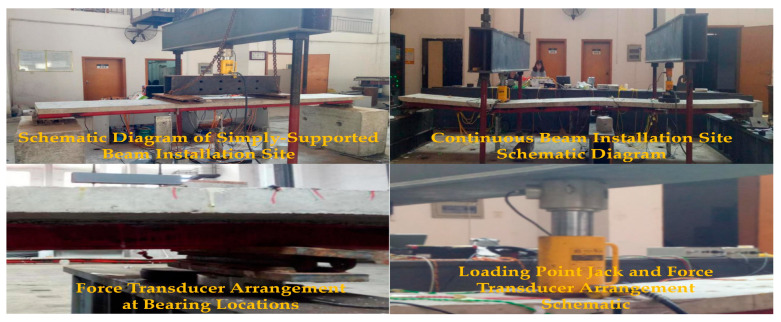
Schematic diagram of specimen installation site and loading point arrangement.

The simply supported beam was subjected to a concentrated loading at midspan, with both end supports and the loading point fixed to the concrete base blocks using mortar. In contrast, the continuous beam was loaded synchronously across two spans. Loads were applied at the midpoints of a 3 m span and a 2 m span, with the load on the 3 m span being approximately 0.7 times that on the 2 m span. The support reactions of the continuous beam were monitored in real time using a spoke-type load cell, as shown in Figure 6. The loading process was controlled in stages: initially, the load was applied in increments of one-tenth of the estimated failure load. When the slope of the load-deflection curve showed a significant downward trend, the loading mode was switched to displacement control, with the jack advancing by 2 mm per increment. Data were continuously recorded until the specimen was completely damaged.

**Figure 7:**
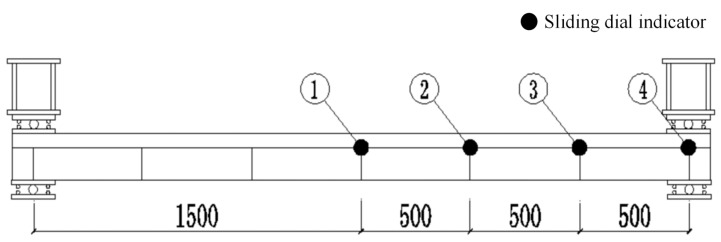
Arrangement of slip measurement points for composite box girder.

The experimental measurement system encompasses multidimensional mechanical responses. The load-deflection curves were obtained using a 500 kN load cell at midspan and spoke-type sensors at the supports of the continuous beam. The interface slip between the steel beam and the concrete slab was measured using slip micrometers located at ① midspan, ② 2 L/3, ③ 5 L/6, and ④ support positions, as detailed in Figure 7. The strain monitoring of the concrete slab included three measuring points on the top surface and three points in the thickness direction, while the strain monitoring of the steel beam covered three points on the web and three points on the bottom flange at midspan. All strain gauges were treated for moisture protection. Throughout the entire test, the development of concrete cracks was documented synchronously, including crack width, cracking load, local buckling phenomena, and residual deformation after failure.

The theoretical calculations were conducted based on the principles of structural mechanics and current standards. The flexural strength of the simply supported beam was calculated using the formula for a single-span beam. For the continuous beam, the calculations strictly followed the methods specified in Code for Design of Steel Structures (GB 50017-2017) [88], with separate computations for the flexural strength in the positive and negative moment regions. The results of these calculations are presented in Table 3.

The results in the table indicate that the midspan moment values are significantly positively correlated with the degree of shear connection. When the cross-sections are approximately equivalent, the influence of different cross-sectional forms on the midspan moment is minimal. The ratio of the moment at the midspan of the 3 m span to the moment at the intermediate support is 1.23, while the corresponding ratio for the 2 m span is 1.17.

The relationship between the midspan moment and the midspan load-carrying capacity can be derived using the methods of structural mechanics, as shown in Equations (2) and (3). The theoretical calculation results for the load-carrying capacity are presented in Table 4.

(2) $$P_{1}=1.814M_{1}+0.32M_{2},$$

(3) $$P_{2}=2.48M_{2}+0.728M_{1},$$

The theoretical calculations in the elastic stage indicate that the ratio of the load-carrying capacities of the two spans is approximately 0.69, which shows good consistency with the experimental load proportion settings.

## 3. Results

### 3.1. Experimental Phenomena

**Figure 8:**
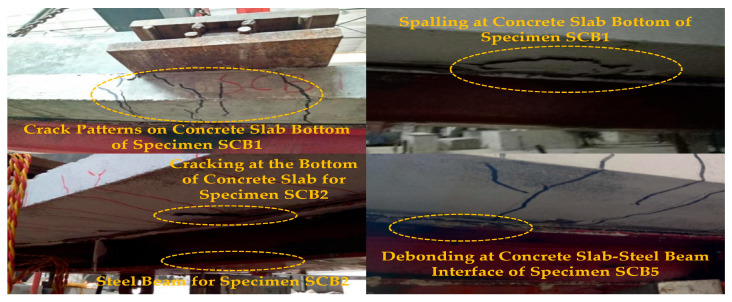
Experimental Phenomena in the Elastic-Plastic Stage of a Simply Supported Composite Beam.

**Figure 9:**
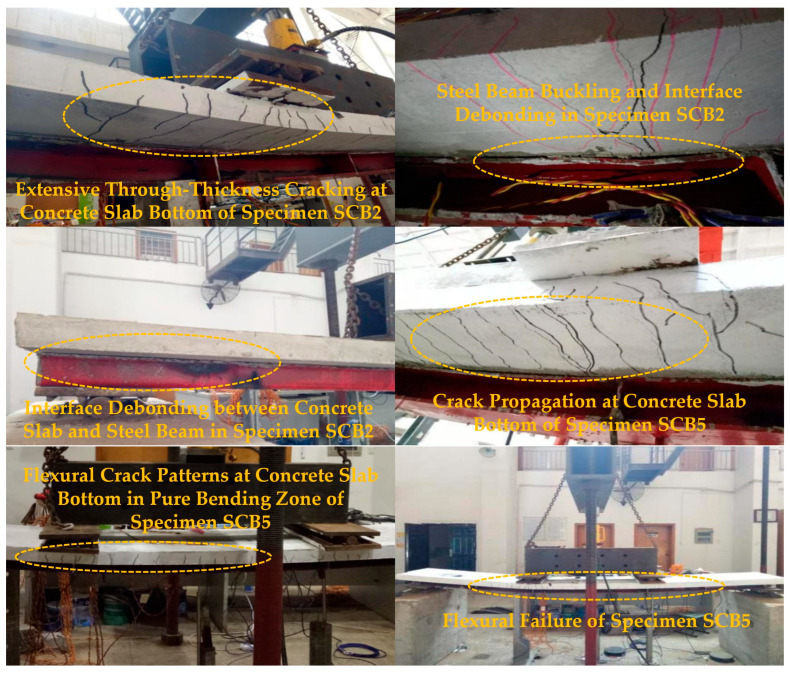
Failure modes of simply supported composite beams.

The static loading tests on six simply supported composite beams after sustained-creep unloading exhibited a clear three-stage response: elastic, elastoplastic, and failure. In the elastic stage, the load-deflection relationship remained linear up to approximately 50% of the ultimate load, and no visible cracks were observed. Upon entering the elastoplastic stage, the curve slope decreased progressively, and loading was switched to displacement control. Flexural cracks initiated at the soffit of the concrete slab and propagated rapidly upward; in specimen SCB1, local crushing and spalling appeared at the slab bottom (Figure 8). Beams SCB2 and SCB5, which had lower degrees of shear connection, produced audible shear-stud ruptures accompanied by abrupt interfacial slip. Multiple through-thickness longitudinal cracks formed along the slab soffit, while dense transverse cracks developed in the negative-moment regions (Figure 8). During the failure stage, mid-span deflection increased sharply, and the load ceased to rise. Fully shear-connected specimens displayed fan-shaped through-cracks, pronounced bottom-flange buckling of the steel beam, and complete interfacial debonding. In contrast, SCB2 and SCB5 developed typical flexural crack bands within the constant-moment region (Figure 9); the maximum crack width reached 4 mm. When the deflection attained 1/30–1/50 of the span, the concrete slab lifted globally and flexural failure ensued.

The static tests on four continuous composite beams after sustained creep again exhibited a distinct three-stage response: elastic, elastoplastic, and failure. In the elastic stage, loads were applied simultaneously to both spans in a 7/10 ratio, accompanied by slight interfacial slip. Cracking initiated directly over the internal support when the load reached 15~20% of the ultimate capacity; the crack width remained below 0.3 mm until the load attained 50% of the ultimate value.

Upon entering the elastoplastic stage, flexural cracks appeared at the soffits of both mid-spans and rapidly propagated through the slab thickness in the negative-moment region with an essentially symmetrical pattern; the crack spacing on the 3 m span side was marginally larger than on the 2 m span side. As detailed in Table 5, the cracking loads for the 3 m span and 2 m span varied across different specimens, with SCB7 experiencing the lowest cracking load of 20 kN in the negative moment region for the 3 m span, and SCB10 showing the highest cracking load of 35 kN for the 2 m span. The average crack spacing also varied, with SCB7 showing the widest spacing at 107 mm and SCB10 the narrowest at 99 mm.

**Figure 10:**
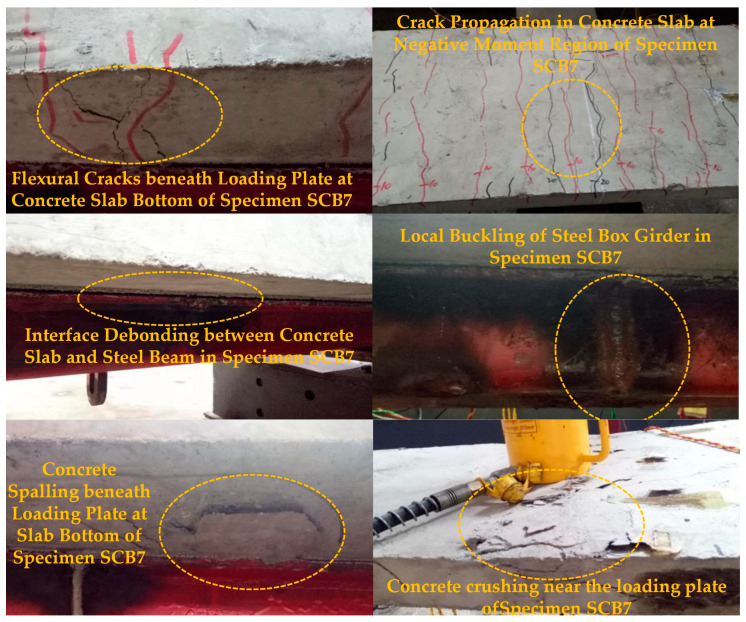
Experimental Phenomena of Continuous Composite Beam Specimens in the Elasto-Plastic Stage.

Local crushing and spalling of the concrete were first observed beneath the loading plates of specimen SCB7, followed by the formation of multiple parallel cracks in the negative-moment region (Figure 10). As the degree of shear connection decreased, interfacial slip accelerated, audible shear-stud ruptures occurred, the bottom flange of the steel girder underwent local buckling, and complete debonding between the concrete slab and the steel beam was evident (Figure 10).

**Figure 11:**
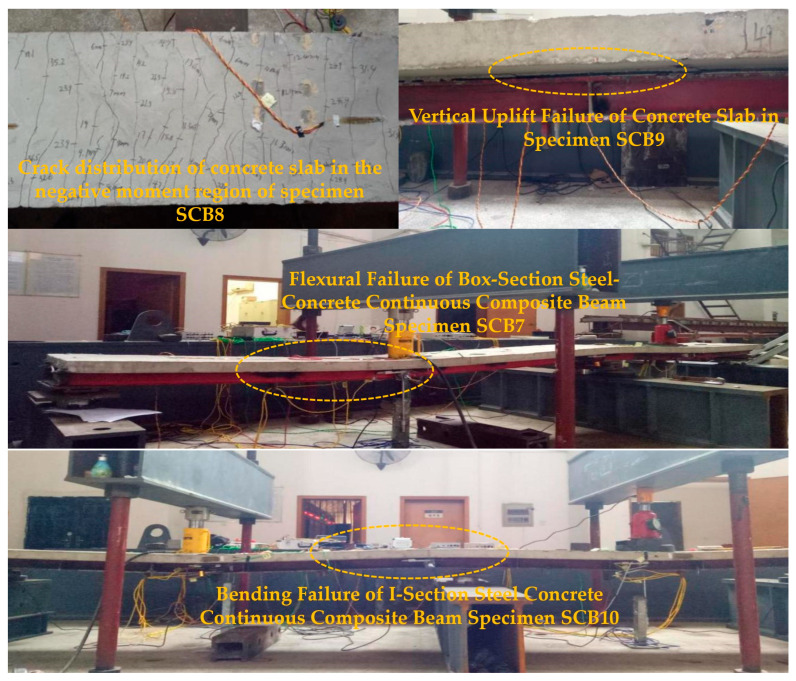
Failure Modes of Continuous Composite Beam Specimens.

During the failure stage, cracks in the negative-moment region continued to widen. In specimens SCB7 and SCB8 with partial shear connection, the maximum crack width reached 4 mm, and the concrete slab exhibited pronounced vertical uplift adjacent to the internal support (Figure 11). Conversely, fully shear-connected specimen SCB10 developed a finer crack pattern with a maximum width not exceeding 3 mm; failure was characterized by local buckling of the steel girder accompanied by global flexural failure of the concrete slab (Figure 11). Loading was terminated once the capacity of either or both spans decreased markedly, signifying the ultimate failure of the continuous composite beams.

### 3.2. Analysis of Experimental Results

This chapter presents a systematic experimental investigation into the mechanical behavior of steel-concrete composite beams. The results demonstrate that the degree of shear connection is a critical factor influencing both load-bearing capacity and stiffness. Specimens with full shear connection exhibited significantly superior performance compared to those with partial connection, while the difference between using a box section and an I-section had limited influence on the overall structural behavior. Creep effects led to an approximate 10% reduction in stiffness; increasing the degree of shear connection effectively enhanced flexural stiffness and mitigated the reduction in concrete compressive strength. Contributions from the concrete slab should be considered in stiffness calculations within the negative moment regions.

Detailed results regarding strain distribution and interfacial slip behavior, comprising extensive data and graphical curves, are provided in Appendix A and Appendix B for further reference.

#### 3.2.1. Stiffness Analysis

1. Stiffness evolution analysis under creep effects

**Figure 12:**
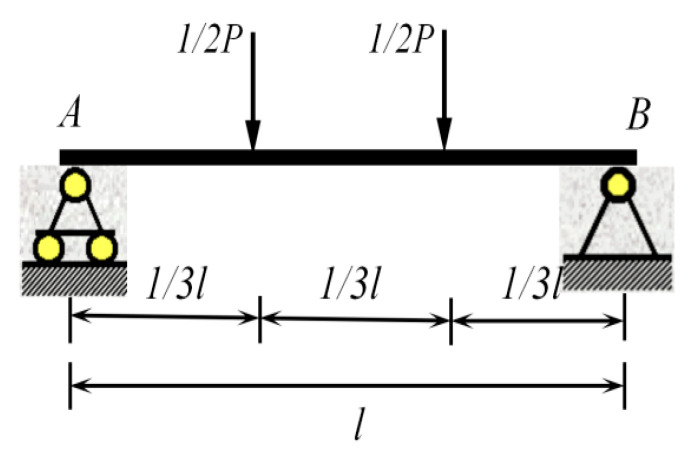
Distribution beam loading.

In the elastic stage, the mid-span deflection of the simply supported beam can be calculated based on the known stiffness. Using the distribution beam loading method (with loads applied at 1/3 and 2/3 of the beam length), as shown in Figure 12.

The mid-span deflection formula, as presented in Equation (4), is derived through the superposition theory, and the measured flexural stiffness is subsequently calculated using Equation (5).

(4) $$\omega =-\frac{23\;Pl^{3}}{1296\;EI},$$

(5) $$EI_{measured}=-\frac{23\;Pl^{3}}{1296\;\omega_{measured}},$$

Based on Nie Jianguo’s stiffness reduction method [35] considering slip and Code for Design of Steel Structures (GB 50017-2017) [88], the reduced stiffness considering slip is calculated, as shown in Equation (6).

(6) $$B=\frac{EI_{eq}}{1+\zeta },$$

where E is the elastic modulus of the steel beam, $I_{eq}$ is the transformed moment of inertia of the composite beam section, and ζ is the stiffness reduction coefficient. The elastic modulus $E_{c}$ in $I_{eq}$ is converted based on the measured cubic compressive strength of concrete. The results in Table 5 show that, for specimens with the same cross-section, the stiffness increases with the shear-connection degree while the stiffness reduction factor decreases accordingly; the I-section beams exhibit a slightly higher stiffness than the box-section beams, and the values listed in column 4 ($EI_{p}$) represent the calculated stiffness obtained by the described method.

**Figure 13:**
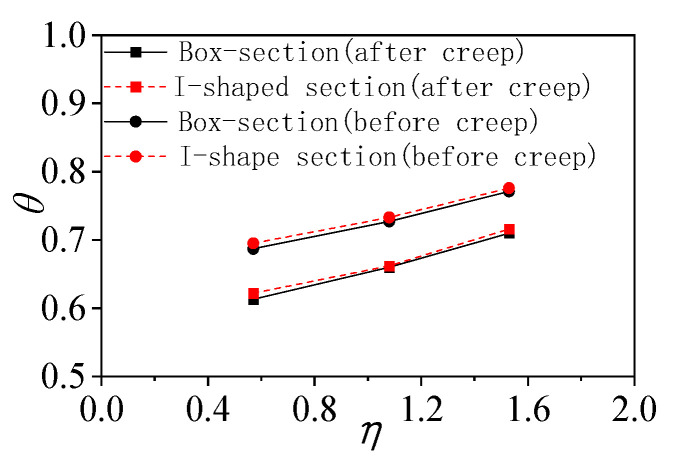
Comparison of θ−η relationships.

The relationship between the measured flexural stiffness and the converted flexural stiffness of the steel-concrete composite beam is illustrated in Equation (7) [90].

(7) $$\left(EI\right)=\theta {\left(E_{s}I\right)}_{0},$$

where $\left(EI\right)$ represents the measured flexural stiffness, θ is the flexural stiffness coefficient, ${\left(E_{s}I\right)}_{0}$ is the converted flexural stiffness of the section without considering creep, $E_{s}$ is the elastic modulus of the steel beam, and I is the converted moment of inertia of the section. Based on the measured deflections of simply supported composite beams, the post-creep measured flexural stiffness $EI_{m}$ and stiffness coefficient $\theta_{m}$, together with their pre-creep calculated counterparts $EI_{c}$ and $\theta_{c}$, are listed in Table 6; the relationship before and after creep is illustrated in Figure 13.

Test results show that creep markedly reduces specimen stiffness by approximately 10%. Increasing the shear-connection degree significantly enhances flexural stiffness: the box-section specimen SCB3 (degree 1.53) exhibits increases of 7.55% and 15.75% over SCB1 (1.08) and SCB2 (0.57), respectively, while the I-section specimen SCB6 (1.53) gains 8.18% and 15.13% compared with SCB4 (1.08) and SCB5 (0.57). At the same connection degree, the difference in flexural stiffness between the two cross-sectional shapes is negligible, indicating that section geometry has a limited influence on stiffness. After the creep tests, the reduced stiffness of the concrete slab lowers the overall stiffness. Using the measured flexural stiffness and a slip-based stiffness-reduction method, the post-creep elastic modulus $E_{c}$ of the concrete is derived, its compressive strength $f_{cu}$ is estimated, and the reduction ratio $R_{f_{cu}}$ is presented in Table 6. The results demonstrate that creep decreases concrete strength, and a higher shear-connection degree leads to a smaller reduction in compressive strength.

2. Correlation between stiffness calculation and deflection in negative moment regions

For continuous composite beams, within the negative moment zone (spanning 0.15 L on either side of the intermediate support), the flexural rigidity of the cross-section considers only the contribution of the longitudinal reinforcement and the steel beam. In the remaining regions, a stiffness reduction method accounting for interface slip effects is employed. According to Code for Design of Steel Structures (GB 50017-2017) [88] and Code for Design of Concrete Structures (GB 50010-2010) [91], Table 7 summarizes two stiffness calculation schemes for the negative moment zone and their corresponding elastic deflection results. The parameters in the table are defined as follows: $\eta^{-}$ is the shear connection degree in the negative moment zone; $EI_{1}$ is the flexural rigidity considering only the contribution of the longitudinal reinforcement and the steel beam; $EI_{2}$ is the flexural rigidity considering the contribution of concrete within an effective slab thickness of $0.6h_{c}$; $\Delta_{0}$ is the measured mid-span deflection under 0.4 times the ultimate load; $\Delta_{1}$ and $\Delta_{2}$ are the calculated elastic-stage mid-span deflections based on $EI_{1}$ and $EI_{2}$, respectively.

Comparative analysis indicates that the deflections of specimens with full shear connection are significantly smaller than those with partial shear connection. Specifically, the deflections of the box-shaped cross-section specimen SCB8 at 3 m and 2 m spans are 10.2% and 21.1% smaller than those of SCB7, respectively. Similarly, the deflections of the I-shaped cross-section specimen SCB10 at the same spans are 9.9% and 21.0% smaller than those of SCB9, respectively. Under the same shear connection degree, the difference in deflection between specimens with box-shaped and I-shaped cross-sections is minimal, indicating that the cross-sectional shape has a limited influence on the deflection in the elastic stage.

The calculated deflection values are generally larger than the measured values $\Delta_{0}$. Among them, the deflection $\Delta_{2}$, calculated based on stiffness $EI_{2}$ (which considers the contribution of concrete within $0.6h_{c}$), is closer to the measured value $\Delta_{0}$ than $\Delta_{1}$. This demonstrates that the cracked concrete slab still contributes partially to the stiffness, and neglecting this contribution in calculations leads to an underestimation of the section stiffness. Therefore, when calculating the flexural rigidity of composite beams in the negative moment zone, it is advisable to refer to Code for Design of Concrete Structures (GB 50010-2010) [91] and include the contribution of the concrete within the effective slab thickness of $0.6h_{c}$.

#### 3.2.2. Load-Bearing Capacity Analysis

1. Influence of shear connection degree

**Figure 14:**
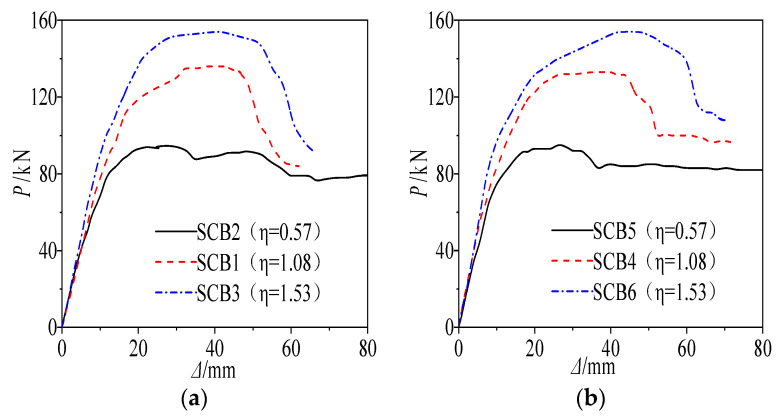
Comparison of Load-Deflection Curves of Steel-Concrete Composite Beams under Different Shear Connection Degrees: (**a**) Test Results of Steel-Concrete Composite Box Girders; (**b**) Test Results of Steel-Concrete Composite I-Girders.

Figure 14 presents the load-deflection curves of the steel-concrete composite beams. The results indicate that the ultimate flexural capacity is positively correlated with the degree of shear connection. Specifically, compared with specimens SCB1 (connection degree 1.53) and SCB2 (0.57), the box-section specimen SCB3 (1.53) exhibited capacity increases of 14.8% and 63.2%, respectively. Likewise, for the I-section specimens, SCB6 (1.53) showed capacity improvements of 15.7% and 62.1% over SCB4 (1.08) and SCB5 (0.57). These observations confirm that beams with full shear connection possess substantially higher load-bearing capacity than those with partial connection, because full shear connection ensures effective composite action between the steel beam and the concrete slab.

2. Effects of cross-sectional configuration

When the specimens possess comparable cross-sectional geometries and identical degrees of shear connection, the disparity in ultimate capacity is negligible. Specifically, at a shear-connection degree of 0.57, the capacities of the two series are identical; at degrees of 1.08 and 1.53, the box-section specimens exceed their I-section counterparts by merely 0.75% and 0.65%, respectively.

3. Theoretical calculation results

**Figure 15:**
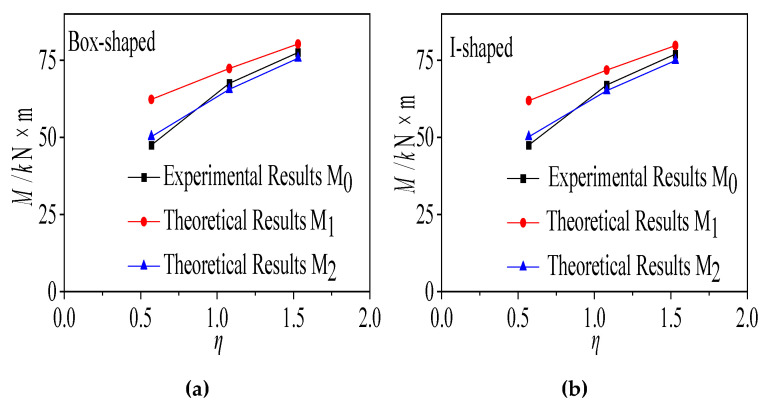
Relationship Curve of Simply Supported Composite Beam Specimens: (**a**) M−η Relationship Curve of Box Section; (**b**) M−η Relationship Curve of I-Section.

Based on Code for Design of Steel Structures (GB 50017-2017) [88], the theoretical ultimate moments $M_{1}$ (without concrete reduction) and $M_{2}$ (with concrete reduction) of simply supported composite beams were calculated and compared with the measured ultimate moment $M_{0}$ (Table 8, Figure 15). For specimens subjected to long-term creep, the theoretical predictions $M_{2}$ are in notably closer agreement with the experimental values, thereby validating the effectiveness of the concrete strength reduction method for estimating the ultimate capacity of composite beams after creep.

**Figure 16:**
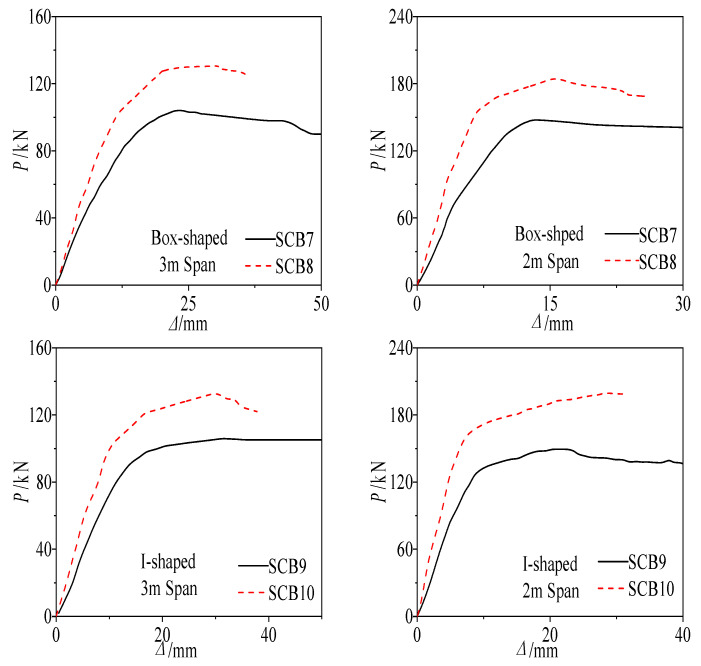
Load-Deflection Curve of Two-Span Continuous Composite Beam Specimens.

The load-deflection curves and flexural capacities of the continuous composite beam specimens are presented in Figure 16 and Table 9. The experimental results demonstrate that the capacity of fully shear-connected specimens markedly exceeds that of partially connected specimens. Specifically, for the box-section specimens, the capacity of SCB8 was 25.96% and 25% higher than that of SCB7 for the 3 m and 2 m spans, respectively. Likewise, for the I-section specimens, the capacity of SCB10 exceeded that of SCB9 by 29.09% and 29.53% for the 3 m and 2 m spans, respectively.

Consequently, when the degree of shear connection is identical, the influence of cross-sectional shape on the ultimate mid-span capacity is negligible; the theoretical ultimate capacities of box-section and I-section specimens are essentially identical.

#### 3.2.3. Deflection Curve Analysis

1. Impact of shear connection degree

**Figure 17:**
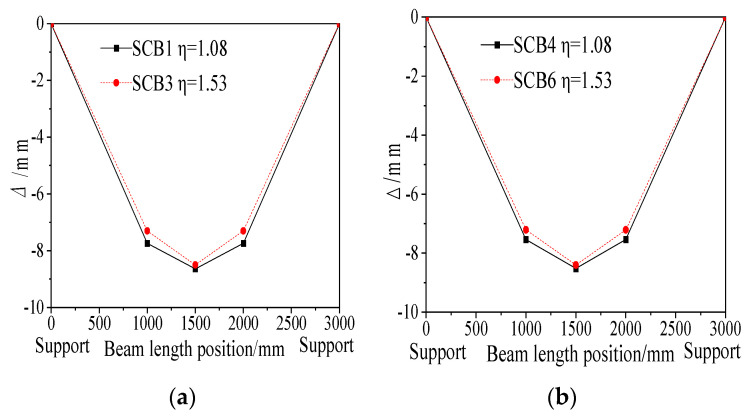
Deflection curves of steel-concrete composite beams with different cross-sectional shapes: (**a**) Box section; (**b**) I-section.

Specimens with partial shear connection exhibit lower load and deflection during the elastic phase due to their reduced load-bearing capacity; for instance, SCB2 and SCB5 demonstrate this behavior and thus cannot be directly compared with fully shear-connected specimens. For fully shear-connected specimens, Figure 17 illustrates the deflection curves at 0.4 times the ultimate load. The results show that under the same loading conditions, the box-section specimen SCB3 has less deflection than SCB1, and the I-section specimen SCB6 has less deflection than SCB4. This indicates that for fully shear-connected specimens, a higher degree of shear connection results in smaller deflection at 0.4 times the ultimate load, suggesting greater flexural stiffness.

2. Influence of cross-sectional form

**Figure 18:**
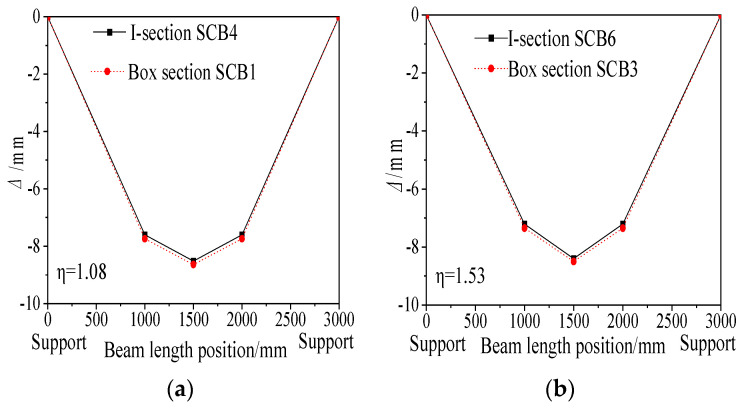
Deflection curves of composite beam specimens with different connection degrees: (**a**) Specimen with a connection degree of 1.08; (**b**) Specimen with a connection degree of 1.53.

Figure 18 compares the deflection curves of composite beam specimens with different cross-sectional shapes. Under the same degree of shear connection, the deflection curves of the box-section specimens SCB1 and SCB3 are nearly identical to those of the I-section specimens SCB4 and SCB6. Specifically, the mid-span deflection of SCB1 is only 1.4% greater than that of SCB4, and the deflection of SCB3 is 1.2% greater than that of SCB6. This negligible difference highlights the limited impact of cross-sectional shape on the mid-span deflection and overall stiffness of the specimens, with the I-section specimens exhibiting slightly less deflection.

## 4. Discussion

This chapter aims to systematically elaborate on the experimental results regarding the flexural behavior of steel–concrete composite beams after creep effects. The discussion focuses on six key dimensions: validation and comparative analysis of results, key parameters and failure mechanisms, experimental error analysis, new insights into basic materials science, novel achievements in engineering applications, and research summary and prospects. By conducting a systematic comparison with existing research findings, this chapter clarifies the theoretical contributions and practical value of the present work.

### 4.1. Verification of Results and Comparative Analysis

To ensure the reliability of the research results, a combined experimental and theoretical approach was adopted for verification:

1. Theoretical Verification: The theoretical ultimate bending moment and stiffness of the specimens were calculated based on the Chinese “Code for Design of Steel Structures” (GB 50017-2017) [88] and the stiffness reduction method considering interface slip proposed by Nie Jianguo et al. [35]. The results show that for specimens subjected to creep, the theoretical ultimate bending moment calculated using the reduced concrete strength (*M*_2_) agrees well with the experimental values (*M*_0_) (Table 7), validating the effectiveness of this method.
2. Inverse Analysis Verification: The post-creep elastic modulus (*E*_c_) and compressive strength (*f_cu_*) of the concrete were determined by back-calculating from the measured load-deflection curves and stiffness, thereby quantifying the degree of material property degradation due to creep (Table 5).

To intuitively illustrate the similarities, differences, and advancements of this study relative to existing works, a simplified comparative table is presented below (Table 10):

Compared with existing studies, the conclusions of this work are both consistent and provide further depth:

1. The shear connection degree (*η*) is key to controlling the long-term performance of composite beams, which aligns with the design principles of Eurocode 4 [34] and the findings of Bradford and Gilbert [63] on the long-term behavior of continuous composite beams.
2. This study systematically quantifies the 40~60% reduction range of the concrete elastic modulus after creep for the first time, providing important experimental data to support the theoretical time-dependent analysis models established by Gilbert [62] and Tarantino & Dezi [61].
3. The finding that cracked concrete in the negative moment region of continuous beams still contributes to stiffness is consistent with the conclusions of Fan Jiansheng et al. [65,66], suggesting that this contribution should be considered in practical design to avoid underestimating stiffness.

### 4.2. Key Parameters and Failure Mechanisms

The failure of steel-concrete composite beams is primarily characterized by steel yielding, concrete crushing, and shear stud slip, which fall under the category of ductile failure. Therefore, fracture toughness $\left(K_{c}\right)$ is not a core indicator for the design or assessment of this type of structure. This study identifies several key parameters affecting their performance, especially after creep:

1. Shear Connection Degree (*η*): This is the most critical parameter. It directly controls the composite action between steel and concrete, playing a decisive role in the beam’s stiffness, load-bearing capacity, and interface slip behavior. The load-bearing capacity of fully shear-connected (*η* = 1.53) specimens was over 60% higher than that of partially connected (*η* = 0.57) specimens and could significantly delay the onset of interface slip.
2. Boundary Conditions: Through internal force redistribution, continuous beams exhibit better crack resistance in the negative moment region and higher mid-span load-bearing capacity than simply supported beams, with a plastic moment redistribution coefficient reaching up to 40%.
3. Creep Effects: Concrete creep leads to significant internal force redistribution, an overall stiffness reduction (approximately 10%), and a substantial decrease in the concrete elastic modulus (40~60%). The extent of this degradation is inversely related to the shear connection degree.
4. Cross-Sectional Form: Due to their better torsional restraint, box-sections exhibited 10~15% lower creep deflections than I-sections, but their influence on ultimate load-bearing capacity and stiffness was much smaller than that of the shear connection degree.

### 4.3. Experimental Errors and Data Analysis

Despite careful experimental design, certain discrepancies exist between measured and theoretical values, primarily due to the following factors:

1. Material Variability: Natural fluctuations in concrete strength and minor variations in stud weld quality are inherent causes.
2. Measurement and Installation Errors: Load eccentricity, support settlement, and strain gauge misalignment can all introduce errors.
3. Theoretical Model Simplifications: The theoretical calculations did not fully simulate the nonlinear development of interface slip or the complex stress state in cracked concrete.

Error analysis of typical specimens (e.g., SCB7 and SCB8) indicates:

1. The measured load-bearing capacity of the partially shear-connected specimen SCB7 was slightly lower than the theoretical value (approximately 93~95% of theoretical). This is likely because its interface slip occurred earlier, limiting the full development of composite action.
2. The experimental data for the fully shear-connected specimen SCB8 agreed well with theoretical values, proving that full connection can effectively suppress detrimental slip.
3. A general trend was that the measured deflections of all specimens in the elastic stage were smaller than the code-calculated values (Table 6). This suggests that current design codes may underestimate the actual stiffness of composite beams, as they do not fully account for the “tension stiffening effect” of cracked concrete.

### 4.4. New Insights into Basic Materials Science

Through systematic experiments, this study has gained new fundamental insights into the interfacial behavior and long-term performance evolution of composite materials:

1. Coupling mechanism between shear connection degree and concrete creep: It is found that a high shear connection degree effectively suppresses the steel-concrete interfacial slip, reduces the stress concentration inside concrete, and thereby delays the degradation rate of its elastic modulus and strength. This reveals the intrinsic correlation between the degree of mechanical connection and the time-dependent degradation of composite component performance, providing an important supplement to the research on composite interfacial behavior.
2. Residual stiffness mechanism of cracked concrete in negative moment regions: Experiments confirm that even after concrete cracking, it can still effectively participate in force transmission within a range of approximately 0.6 times the slab thickness and provide continuous stiffness contribution. This finding revises the traditional simplified assumption that “cracked concrete completely ceases to work” and improves the basic theory for stiffness calculation of composite materials.
3. Quantitative law of creep-induced stiffness degradation in composite beams: This study quantifies for the first time that “the overall stiffness decreases by approximately 10% after 180 days of creep” and clarifies the quantitative relationship that “for every 0.5 increase in shear connection degree, the stiffness degradation rate can be reduced by 8–10%”. This provides key experimental data support for the development of more accurate long-term performance prediction models for composite materials.

### 4.5. Novel Achievements in Engineering Applications

Based on the aforementioned research, this study proposes the following design recommendations and achievements that can be directly applied to engineering practice:

1. Optimized design criteria for shear connection degree: For projects with strict requirements on long-term deflection control (e.g., bridges, long-span floor systems), an ultra-full shear connection design with a shear connection degree of 1.53 is recommended. This measure can reduce creep-induced deflection by 15–20% and increase the strength retention rate to over 55%. For cost-sensitive projects, a full shear connection design with a shear connection degree of 1.08 can be adopted to balance performance and economy.
2. Guidelines for cross-sectional form selection: Box sections are suitable for scenarios with strict restrictions on creep-induced deflection (e.g., floors of precision industrial plants), while I-sections can be used in scenarios with relatively relaxed deflection requirements (e.g., secondary beams of industrial plants). Given that the difference in ultimate bearing capacity between the two is only 0.65%, engineers can make selections based on specific deformation control requirements.

### 4.6. Research Summary and Outlook

Through systematic experimentation, this study quantified the degradation patterns of stiffness and load-bearing capacity in steel-concrete composite beams after creep and established the dominant role of the shear connection degree in controlling creep effects. The proposed calculation method considering the reduction in concrete properties after creep provides an important basis for the long-term performance assessment and design of similar structures.

A limitation of this study is that it did not consider the coupling effects of cyclic loading or extreme environments with creep. Future research could focus on performance evolution under complex service conditions and develop more refined numerical models to simulate interface behavior under long-term loading.

## 5. Conclusions

This study provides a comprehensive experimental investigation into the creep behavior of steel-concrete composite beams, emphasizing the impacts of shear connection degree, cross-sectional form, and boundary conditions. Key findings are as follows:

1. Increased shear connection degrees (1.53) led to a 15~20% reduction in creep-induced deflections compared to partial connections (0.57), indicating enhanced composite action under long-term loading. Full shear connections delayed interfacial slip initiation to approximately 30% of load capacity and maintained strain continuity up to 0.6 times the ultimate load. In contrast, partial connections showed early slip-induced strain discontinuities.
2. Box sections demonstrated 10~15% lower creep deflections than I-sections under identical loading, due to their superior torsional restraint on concrete slab deformation. When shear connection degrees were equivalent, both cross-sectional forms exhibited comparable patterns of creep-induced stiffness reduction (40~60%), suggesting that cross-sectional form plays a secondary role in creep resistance.
3. Continuous beams exhibited a 25% higher cracking load in negative moment regions and a 1.5-fold increase in mid-span moments compared to simply supported beams, due to creep-induced moment redistribution. The plastic moment redistribution coefficient reached 40%, aligning with Eurocode recommendations and highlighting creep’s significant role in internal force redistribution.
4. Post-creep concrete showed a 40~60% reduction in elastic modulus, with the degradation extent inversely related to the shear connection degree. The improved stiffness reduction method effectively quantified residual performance, revealing that fully connected specimens retained 55~61% of their strength, compared to 43~49% for partially connected specimens.

These findings provide critical insights for long-term performance prediction, demonstrating that shear connection design is more crucial than cross-sectional optimization for mitigating creep effects. The quantified 40~60% reduction in modulus aligns with practical observations and should be used to correct overestimations in previous design guidelines. Additionally, special attention must be paid to creep-induced moment redistribution in negative moment regions of continuous systems. The experimental and analytical framework established in this study offers a reliable reference for durability design under sustained loading, while future research should focus on the interactions between creep and cyclic loads, as well as environmental coupling effects.

## Figures and Tables

**Table 1:** Nomenclature.

Symbol	Description	Unit
η	Degree of shear connection	-
$\eta_{r}$	Actual number of shear connectors provided	piece
$\eta_{f}$	Number of shear connectors required for full shear connection design	piece
$f_{c}$	Axial compressive strength	MPa
$f_{cu}$	Cube Compressive Strength of Concrete	MPa
$E_{c}$	Elastic modulus of concrete	MPa
$E_{s}$	Elastic modulus of steel beam	MPa
E	Elastic Modulus of Steel Beam	MPa
σ	Stress	MPa
ε	Strain	-
L	Net span length of composite beam	mm
l	Calculated span of composite beam	mm
$\eta^{+}$	Degree of shear connection in positive moment region	-
$\eta^{-}$	degree of shear connection in negative moment region	-
$M_{0}$	Ultimate bending moment at intermediate support (continuous beam)	kN·m
$M_{1}$	Ultimate bending moment at mid-span of 3 m span (continuous beam)	kN·m
$M_{2}$	Ultimate bending moment at mid-span of 2 m span (continuous beam)	kN·m
$P_{1}$	Ultimate bearing capacity at mid-span of 3 m span (continuous beam)	kN
$P_{2}$	Ultimate bearing capacity at mid-span of 2 m span (continuous beam)	kN
P	Load	kN
$P_{u}$	Ultimate load	kN
ω	Deflection	mm
$\omega_{measured}$	Measured Deflection	mm
I	Moment of Inertia	mm^4^
$I_{measured}$	Measured Moment of Inertia	mm^4^
$I_{eq}$	Transformed section moment of inertia of composite beam	mm^4^
B	Reduced stiffness	10^6^ N·mm^2^
ζ	Stiffness reduction coefficient	-
EI	Calculated stiffness value before creep	10^6^ N·mm^2^
$EI_{p}$	Calculated stiffness of the composite beam	10^6^ N·mm^2^
$EI_{m}$	Post-creep measured flexural stiffness	10^6^ N·mm^2^
$EI_{c}$	Pre-creep calculated flexural stiffness	10^6^ N·mm^2^
$\left(EI\right)$	Measured flexural stiffness of steel-concrete composite beam	10^6^ N·mm^2^
${\left(E_{s}I\right)}_{0}$	Transformed flexural stiffness of section considering creep	10^6^ N·mm^2^
$EI_{1}$	Flexural stiffness calculated in accordance with Code for Design of Steel Structures (GB 50017-2017) [63]	10^6^ N·mm^2^
$EI_{2}$	Flexural stiffness calculated with reference to provisions in Code for Design of Concrete Structures (GB50010-2010) [66]	10^6^ N·mm^2^
θ	Flexural stiffness coefficient	-
$\theta_{m}$	Post-creep stiffness coefficient	-
$\theta_{c}$	Pre-creep stiffness coefficient	-
$R_{f_{cu}}$	Reduction factor of cubic compressive strength of concrete	-
$\Delta_{0}$	Measured deflection corresponding to 0.4 times the ultimate load	mm
$\Delta_{1}$	Theoretical deflection calculated with ${\left(EI\right)}_{1}$ as the stiffness in the negative moment region	mm
$\Delta_{2}$	Theoretical deflection calculated with ${\left(EI\right)}_{2}$ as the stiffness in the negative moment region	mm
$h_{c}$	Thickness of concrete slab	mm

**Table 2:** Parameters of Simply Supported and Continuous Composite Beam Specimens.

Supporting Method		Simply Supported Composite Beams						Continuous Composite Beams			
Specimen		SCB-1	SCB-2	SCB-3	SCB-4	SCB-5	SCB-6	SCB-7	SCB-8	SCB-9	SCB-10
Steel Beam Section Form		Box-shaped cross-section			I-shaped cross-section			Box-shaped cross-section		I-shaped cross-section	
Number of Studs		34	18	48	34	18	48	34	80	34	80
Stud Spacing (mm)		180	310	125	180	310	125	310	125	310	125
Shear Connection Degree	Positive Moment Region	1.08	0.57	1.53	1.08	0.57	1.53	0.57	1.53	0.57	1.53
	Negative Moment Region							0.85	1.68	0.85	1.68
Span Length (mm)		3000						5000			
Concrete Slab Width (mm)		550									
Concrete Slab Thickness (mm)		60									
Number of Stirrups		32Φ8@100						50Φ8@100			
Number of Longitudinal Reinforcing Bars		6Φ8@100						6Φ8@100 (Dense arrangement in the support zone)			

**Table 3:** Calculation of the ultimate bending moment for continuous composite beam specimens.

Steel Beam Section Form	Specimen	Shear Connection Degree		Calculated Value of Ultimate Bending Moment (kN.m)			Ultimate Bending Moment Ratio	
		$\eta^{+}$	$\eta^{-}$	Midspan of 3 m Span M1	Mid-Support M0	Midspan of 2 m Span M2	$\left|M_{1}/M_{0}\right|$	$\left|M_{2}/M_{0}\right|$
Box Section	SCB7	0.57	0.85	51.91	−42.27	49.25	1.23	1.17
	SCB8	1.53	1.68	65.8	−53.49	62.45	1.23	1.17
I-Section	SCB9	0.57	0.85	52.03	−42.31	49.33	1.23	1.17
	SCB10	1.53	1.68	66.16	−53.68	62.62	1.23	1.17

**Table 4:** Calculated bearing capacity of continuous composite beam specimens.

Steel Beam Section Form	Specimen	Calculated Value of Bearing Capacity (kN)		Bearing Capacity Ratio
		Midspan of 3 m Span *P*_1_	Midspan of 2 m Span *P*_2_	*P*_1_/*P*_2_
Box Section	SCB7	109.92	159.93	0.69
	SCB8	139.35	202.78	0.69
I-Section	SCB9	110.17	160.22	0.69
	SCB10	140.05	203.46	0.69

**Table 5:** Cracking Load and Crack Spacing of Continuous Composite Beam Specimens.

Steel Beam Section Form	Specimen	Cracking Load in the Negative Moment Region (kN)		Cracking Load for the 3 m Span (kN)	Cracking Load for the 2 m Span (kN)	Average Crack Spacing (mm)
		3 m Span	2 m Span			
Box Section	SCB7	20	30	52	74	107
	SCB8	25	35	61	90	98
I-Section	SCB9	20	30	53	76	105
	SCB10	25	35	62	93	99

**Table 6:** Comprehensive calculation results of stiffness and concrete strength for composite beam specimens.

**Specimen**	***η***	**ζ**	***EI_p_* (10^6^ N·mm^2^)**	***EI_m_* (10^6^ N·mm^2^)**	***θ_m_***
SCB1	1.08	0.375	2.584	2.342	0.660
SCB2	0.57	0.455	2.442	2.176	0.613
SCB3	1.53	0.297	2.738	2.519	0.710
SCB4	1.08	0.363	2.612	2.371	0.662
SCB5	0.57	0.439	2.475	2.228	0.622
SCB6	1.53	0.288	2.764	2.565	0.716
**Specimen**	***EI_c_* (10^6^ N·mm^2^)**	***θ_c_***	***E_c_* (MPa)**	***f_cu_* (MPa)**	***R_f_cu__* (%)**
SCB1	2.584	0.727	25,394	19.1	55.36
SCB2	2.442	0.687	24,331	16.8	48.70
SCB3	2.738	0.771	26,293	21.2	61.45
SCB4	2.612	0.734	25,080	18.4	53.33
SCB5	2.475	0.695	23,481	15.1	43.77
SCB6	2.764	0.776	25,962	20.4	59.13

**Table 7:** Comprehensive analysis of deflection and concrete strength for composite beams in the elastic stage.

Specimen	$\eta^{-}$	$\begin{array}{c} EI_{1}\\ \left(10^{6}\;N\cdot {\mathrm{mm}}^{2}\right) \end{array}$	$\begin{array}{c} EI_{1}\\ \left(10^{6}\;N\cdot {\mathrm{mm}}^{2}\right) \end{array}$	3 m Span Deflection (mm)			2 m Span Deflection (mm)			3 m Span Deflection (mm)		2 m Span Deflection (mm)	
				$\Delta_{0}$	$\Delta_{1}$	$\Delta_{2}$	$\Delta_{0}$	$\Delta_{1}$	$\Delta_{2}$	$\Delta_{1}/\Delta_{0}$	$\Delta_{2}/\Delta_{0}$	$\Delta_{1}/\Delta_{0}$	$\Delta_{2}/\Delta_{0}$
SCB7	0.875	1.851	2.094	5.29	5.38	5.31	3.41	3.5	3.43	1.017	1.004	1.026	1.006
SCB8	1.75	2.041	2.313	4.75	4.82	4.77	2.69	2.74	2.7	1.015	1.004	1.019	1.004
SCB9	0.875	1.926	2.162	5.07	5.18	5.1	3.14	3.2	3.15	1.022	1.006	1.019	1.003
SCB10	1.75	2.119	2.381	4.57	4.65	4.59	2.48	2.53	2.49	1.018	1.004	1.020	1.004

**Table 8:** Theoretical Calculation Results of Ultimate Moment for Composite Beam Specimens.

Specimen	Shear Connection Degree *η*	Measured Ultimate Moment	Theoretical Calculated Ultimate Moment		Ultimate Moment Reduction Factor
		$M_{0}\left(kN\right)$	$M_{1}\left(kN\right)$	$M_{2}\left(kN\right)$	$M_{2}/M_{1}$
SCB1	1.08	67.5	72.33	65.53	0.907
SCB2	0.57	47.5	62.31	50.24	0.806
SCB3	1.53	77.5	80.27	75.62	0.942
SCB4	1.08	67	71.81	65.12	0.907
SCB5	0.57	47.5	61.93	50.19	0.810
SCB6	1.53	77	79.78	74.89	0.939

**Table 9:** Measured Ultimate Bearing Capacity of Continuous Composite Beam Specimens.

Steel Beam Section Form	Specimen	Measured Ultimate Bearing Capacity (kN)		Ratio to Theoretical Bearing Capacity	
		3 m Span	2 m Span	3 m Span	2 m Span
Box-shaped cross-section	SCB7	104	148	0.95	0.93
	SCB8	131	185	0.94	0.91
I-shaped cross-section	SCB9	106	149	0.96	0.93
	SCB10	133	193	0.95	0.95

**Table 10:** Comparison of Creep-Induced Flexural Performance of Composite Beams: This Study vs. Others.

Author (Year)	Specimen Type	Core Variables	Key Post-Creep Indicators	Main Conclusions	This Study’s Advantages
Fan et al. (2010) [65,66]	Simply supported/cantilever steel-concrete composite beams	Positive/negative bending	3-year deflection = 2.5× initial; negative moment cracking reduces stiffness	Creep/shrinkage increase deflection; cracking must be considered	Adds box/I-section comparison; quantifies shear connection’s creep inhibition
Gilbert & Bradford (1995) [63]	Two-span continuous steel-concrete composite beams	Shrinkage/creep	340-day deflection increased; internal force redistribution driven by shrinkage	Shrinkage dominates creep-period force redistribution	Studies shear connection × section form coupling; provides clearer parameter rules
Nan et al. (2023) [92]	Two-span continuous steel-concrete composite beams	Slip-creep coupling	728-day deflection +14%; steel beam moment +12%	Slip-creep coupling aggravates deflection; higher shear stiffness mitigates creep	Supplements simply supported/continuous comparison; clarifies negative moment cracking protection
Wang et al. (2025) [93]	UHPC/ECC/RC composite beams	Interface roughness, composite layer thickness	Flexural capacity +10–40%; interface bond strength enhanced	Optimizing interface/layer thickness improves creep-period load-bearing capacity	Focuses on classic steel-concrete system (vs. UHPC/ECC); longer test period (3-year data); parameters more engineering-oriented
This Study	Simply supported/continuous steel-concrete composite beams (box/I-section)	Shear connection, section form	Full shear connection reduces creep deflection by 15–20%; box-section < I-section by 10–15%	Shear connection is key to creep inhibition; negative moment reinforcement improves crack resistance	Multi-variable coupling tests; quantifies parameter weights; offers direct engineering design suggestions

**Figure A1:**
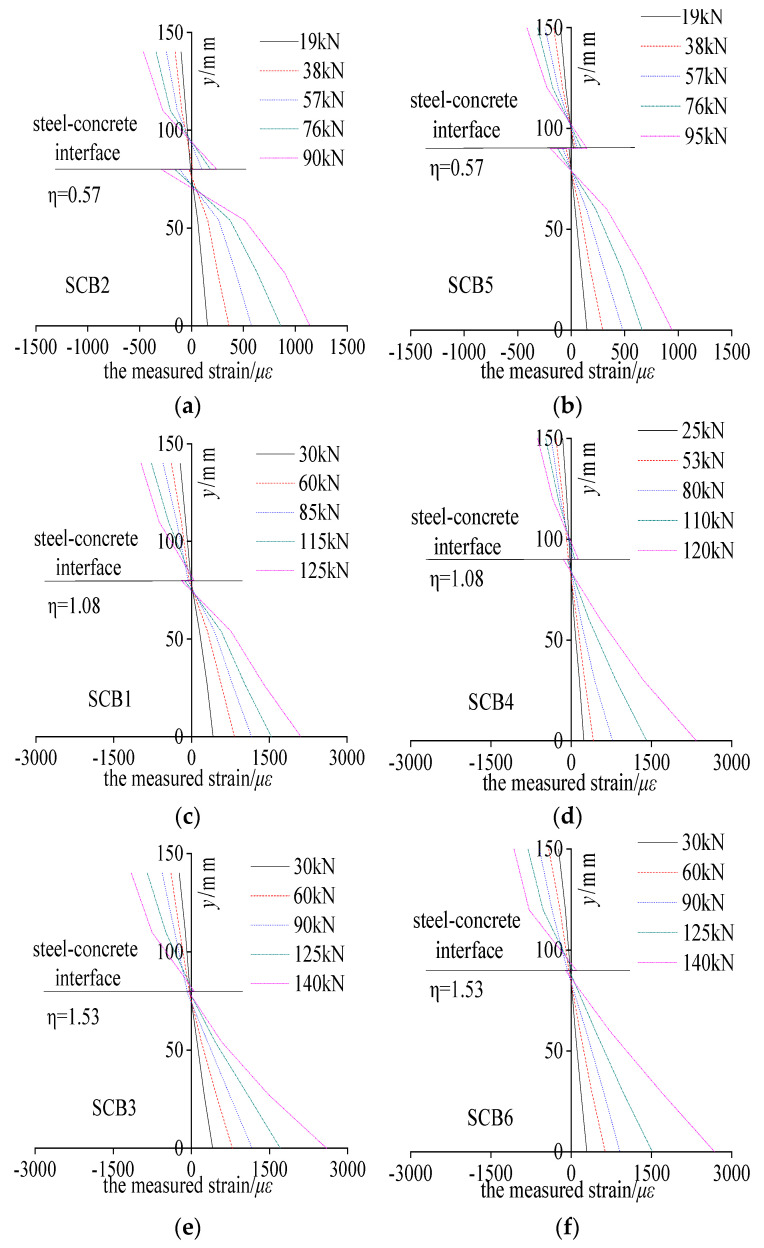
Strain distribution curve at mid-span of the simply supported beam specimen: (**a**) Box-shaped composite beam SCB2; (**b**) I-section composite beam SCB5; (**c**) Box-shaped composite beam SCB1; (**d**) I-section composite beam SCB4; (**e**) Box-shaped composite beam SCB3; (**f**) I-section composite beam SCB6.

**Figure A2:**
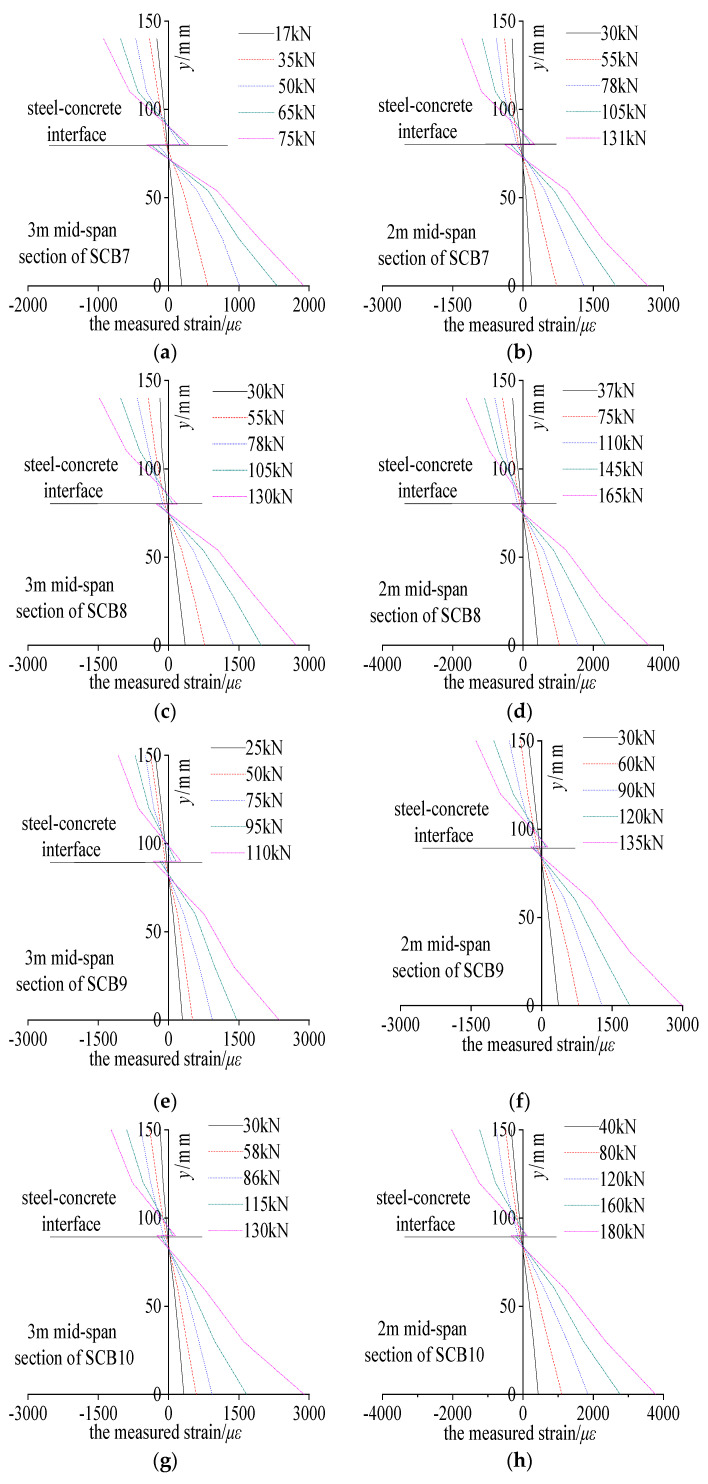
Strain distribution curves at mid-span of the two-span continuous composite beam specimen: (**a**) 3 m mid-span of box-section SCB7; (**b**) 2 m mid-span of box-section SCB7; (**c**) 3 m mid-span of box-section SCB8; (**d**) 2 m mid-span of box-section SCB8; (**e**) 3 m mid-span of I-section SCB9; (**f**) 2 m mid-span of I-section SCB9; (**g**) 3 m mid-span of I-section SCB10; (**h**) 2 m mid-span of I-section SCB10.

**Figure A3:**
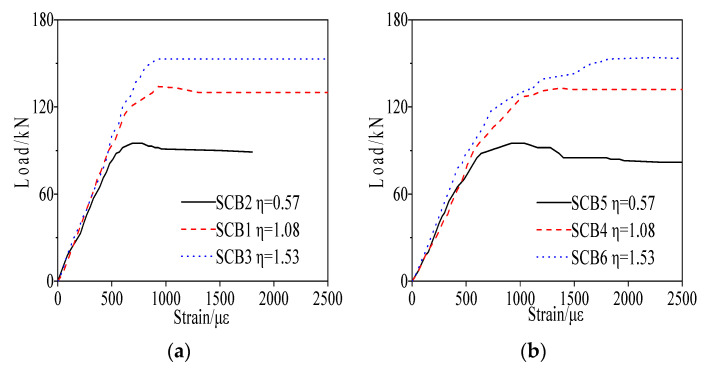
Load-Strain curve at the mid-span bottom flange of the steel beam specimen: (**a**) Box composite beam specimen; (**b**) I-section composite beam specimen.

**Figure A4:**
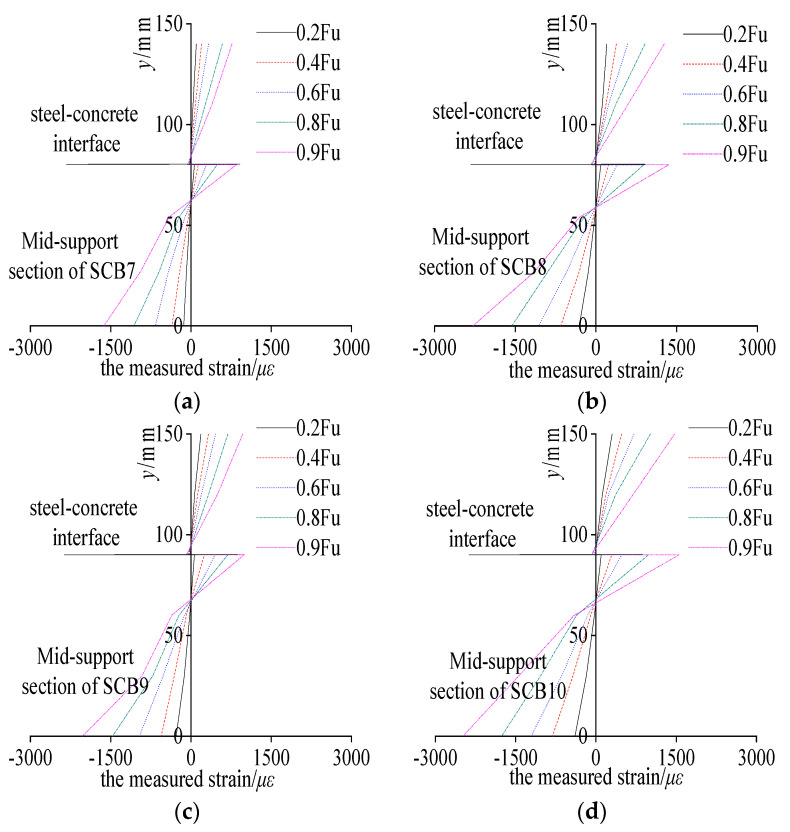
Strain distribution curve at the mid-support section of the continuous composite beam specimen: (**a**) Mid-support of box-section SCB7; (**b**) Mid-support of box-section SCB8; (**c**) Mid-support of I-section SCB9; (**d**) Mid-support of box-section SCB10.

**Figure A5:**
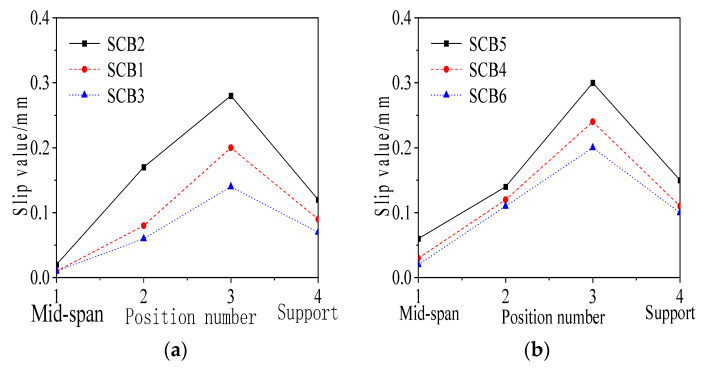
Measured slip curve of simply supported composite beam specimens: (**a**) box-section composite beam; (**b**) I-section composite beam.

**Figure A6:**
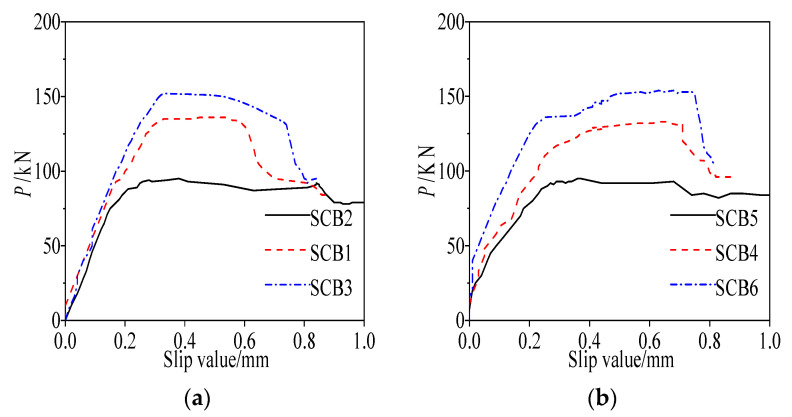
Measured beam-end slip curve of composite specimens: (**a**) box-section composite beam; (**b**) I-section composite beam.

**Figure A7:**
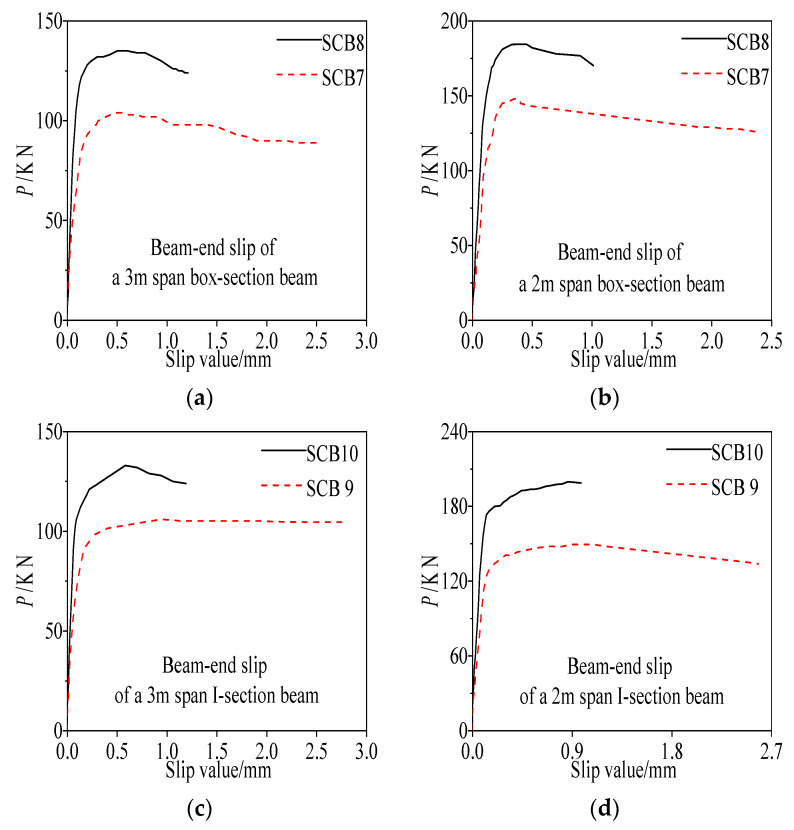
Beam-end slip curve of continuous composite beam specimen. (**a**) Beam-end slip of a 3 m span box-section beam; (**b**) Beam-end slip of a 2 m box-section beam; (**c**) Beam-end slip of a 3 m span I-section beam; (**d**) Beam-end slip of a 2 m span I-section beam.

## Data Availability

The original contributions presented in this study are included in the article. Further inquiries can be directed to the corresponding author.

## Appendix A

### Strain Distribution

1. Strain distribution patterns at mid-span cross-sections

As shown in Figure A1 and Figure A2, the strain profiles satisfy the plane-section hypothesis for simply supported beams when the applied load is below $0.4P_{u}$. With further loading, interfacial slip causes abrupt strain changes; specimens with insufficient shear connection develop double neutral axes at an earlier stage. Fully connected beams retain linear strain distributions up to $0.6P_{u}$. Continuous beams also conform to the hypothesis initially, but it ceases to be valid in the later loading stages owing to progressive slip and plasticity.

2. Strain development characteristics in steel beam bottom flanges at mid-span

As illustrated in Figure A3, the mid-span response of the steel bottom flange in simply supported beams is essentially insensitive to cross-sectional shape. In the initial stage, all specimens exhibit linear strain growth with comparable magnitudes. Once the load reaches approximately $0.5P_{u}$, yielding initiates in the box-section bottom flange, marking the transition to the elastoplastic phase; strains thereafter increase monotonically until failure. The I-section counterparts display an almost identical load-strain response, confirming the negligible influence of sectional configuration on this behavior.

3. Strain distribution features at intermediate support sections

As depicted in Figure A4, the strain distribution along the section height is markedly nonlinear and deviates significantly from the plane-section hypothesis. The concrete slab, subjected to tension, progressively cracks and extends with increasing load. In contrast, the strain profile within the steel beam remains essentially linear and fully satisfies the plane-section assumption.

## Appendix B

### Interfacial Slip Behavior

Under 0.4 times the ultimate load, the relative slip distribution between the concrete slab and steel beam in the simply supported composite beams demonstrates that the maximum slip occurs at a position 1/6 L from the support (Position ③), as provided in Table A1. The slip values exhibit a negative correlation with the degree of shear connection. Although differences in maximum slip values across cross-sectional forms are relatively small, the box section shows slightly lower slip than the I-section. Specimens with incomplete shear connection (SCB2 and SCB5) exhibit end slip exceeding 3 mm. During loading, increasing load leads to accelerated slip, accompanied by shear stud fracture and bond surface separation, as illustrated in Figure A5 and Figure A6, resulting in reduced composite action and subsequent load decrease.

**Table A1:** Measured slip value at Position ③ of the test specimen.

Shear Connection Degree *η*	Box-Section Specimen	Slip Value at Position ③ (mm)	Section Specimen	Slip Value at Position ③ (mm)
1.08	SCB1	0.21	SCB4	0.24
0.57	SCB2	0.28	SCB5	0.31
1.53	SCB3	0.14	SCB6	0.20

For continuous composite beams, specimens with incomplete shear connection (SCB7 and SCB9) show significantly larger beam-end slip compared to those with complete shear connection (SCB8 and SCB10), with detailed values provided in Table A2. In the later loading stage, specimens with incomplete shear connection experience vertical uplift exceeding 10 mm, while those with complete connection exhibit uplift not exceeding 2 mm, indicating superior composite performance of the latter. The corresponding beam-end slip behavior is further depicted in Figure A7.

**Table A2:** Maximum beam-end slip value of continuous composite beam specimen.

Steel Beam Section Form	Specimen	Maximum Beam-End Slip of a 3 m Span Beam (mm)	Maximum Beam-End Slip of a 2 m Span Beam (mm)
Box section	SCB7	2.55	2.36
	SCB8	1.21	1.01
I section	SCB9	2.75	2.58
	SCB10	1.19	0.98
